# What are family caregivers’ experiences of coordinating end-of-life care at home? A narrative review

**DOI:** 10.1017/S1478951524001895

**Published:** 2025-01-24

**Authors:** Harriet Nicholls, Matthew Carey, Kevin Hambridge

**Affiliations:** 1 School of Nursing and Midwifery, University of Plymouth, Plymouth, UK; 2 School of Health and Social Wellbeing University of the West of England, Bristol UK

**Keywords:** Family caregiver experience, care coordination, end-of-life, palliative, home setting

## Abstract

**Objectives:**

People with life-limiting diseases, who are no longer receiving active or curable treatment, often state their preferred place of care and death as the home. This requires coordinating a multidisciplinary approach, using available health and social care services to synchronize care. Family caregivers are key to enabling home-based end-of-life support; however, the 2 elements that facilitate success – coordination and family caregiver – are not necessarily associated as being intertwined or one and the same. This narrative review explores family caregiver experiences of coordinating end-of-life care in the home setting.

**Methods:**

Studies were identified systematically following the Preferred Reporting Items for Systematic reviews and Meta-Analyses (PRISMA) guidelines. A search of 5 databases (CINAHL, AMED, MEDLINE, Joanna Briggs Institute for Systematic Reviews, and the Cochrane Database) was conducted using Medical Subject Headings search terms and Boolean operators. Seven hundred and eighty papers were screened. Quality assessment was conducted using the JBI Critical Appraisal Checklist for Qualitative Research. Characteristics of included studies were extracted using the Joanna Briggs Institute Qualitative Assessment and Review Instrument (JBI-QARI) extraction tool.

**Results:**

Ten qualitative studies were included. A meta-aggregative approach was used to assemble findings and categories extracted from the included papers, which led to identification of 3 overall themes: (1) family caregiver identity, (2) strategies for care, and (3) practicalities of care.

**Significance of results:**

Research suggests there should be a designated care coordinator to support people nearing the end of life at home. However, this review shows that family caregivers intrinsically take on this role. Their experiences, frequently share commonalities across different cultures and regions, highlighting the universal nature of their challenges. Difficulties associated with providing home-based care are evident, but the undertaking of care coordination by relatives highlights a need for a change in approach. Future studies could explore the impact of having a designated “facilitator” or single point of contact for families, as well as the development of tailored communication strategies.

## Introduction

In recent decades, there has been a notable shift in healthcare from the paternalistic approach toward emphasizing patient-centered care and respecting the autonomy and preferences of individuals facing the end of life (Taylor 2009). In the United Kingdom (UK), this is epitomized by the Ambitions for Palliative and End of Life Care national framework (National Partnership for Palliative and End of Life Care 2021). This sets out 6 ambitions for all those supporting a person, written in the voice of the dying person and placing them at the center of these statements. The ambitions also consider the support and well-being of family, recognizing the physical and emotional care they provide, with the intention of alleviating burden. Consequently, more individuals express their desire to receive end-of-life care in the comfort and familiarity of their homes, surrounded by loved ones (Grande et al. 2021). This preference has placed family caregivers at the forefront of the caregiving process, assuming roles that involve physical, emotional, and logistical support. Family caregivers often feel intense responsibility for the overall care of their loved one, which can impact upon the physical, emotional, and mental health of caregivers (Stajduhar et al. 2010).

Palliative care is a broad approach focusing on improving quality of life for an individual with serious, life-limiting illness; end-of-Life care focuses on care in the final phase of life. This care is geared toward managing symptoms, providing emotional support, and ensuring dignity for patients in their final days, often involving family members and caregivers. Both address the physical, emotional, and spiritual needs of the patient, aiming to enhance overall well-being, with end-of-life care attending more closely to preparing for death and supporting the dying process.

Research on family caregiving has identified themes that interlink coordination of care, the burdens felt, the barriers and challenges to providing care in the home setting and the potential support needs of caregivers. Penrod et al. (2012) explored the caregiving process and defines the basic social approach as “seeking normality,” as caregivers strive to establish reliable patterns of everyday life while fulfilling the demands of end-of-life care. The recognition of caregivers’ efforts in maintaining a sense of normalcy underscores the coordination role they play in ensuring a smooth transition during this challenging period while recognizing the heavy burden upon them as family caregivers (Bijnsdorp et al. 2022).

Family caregiving involves providing practical, emotional, or educational support to individuals of all ages with various health needs (Stall et al. 2019). Family caregivers, often close relatives or friends, play a crucial role in palliative care, which aims to enhance quality of life by managing symptoms and offering emotional support (Mulcahy Symmons et al. 2022; WHO 2020). Despite the fact that over 56 million people worldwide need palliative care annually, only 14% receive it due to barriers such as lack of integration into health systems and inadequate training and medication access (WHO 2020).

In the context of the UK, the demand for palliative care is rising with an aging population. By 2050, 1 in 4 people will be 65 or older (ONS 2019). Family caregivers account for 75% of home-based care being provided to people in the UK and, within end-of-life care this number can rise to 90% (Gardiner et al. 2020).

It is recognized that for many, home is the preferred place of death (Fereidouni et al. 2021; Woodman et al. 2016), while actual place of death can be changeable due to a number of factors (Ho et al. 2022). This can vary significantly across different cultures and societies, but in an umbrella review that encompassed studies from Europe, North America, Asia, Latin America, Oceania and Africa, Pinto et al. (2023) established that home is most preferred choice for place of death in all studies. Families can greatly influence place of death, with willingness to provide the care and family size being facilitating factors (Ho et al. 2022; Sayma et al. 2020). This invaluable unpaid workforce enables people to be palliated in their preferred place of care, which in many cases would otherwise be unachievable. The World Health Organization (2020) supports the concept that a wide range of services and professionals should facilitate delivery of palliative care, in support of patient and family. But the reality remains less assimilated.

Family caregivers often mediate between health and social care professionals, navigating complex care options, budgets, and payments, which can be stressful and financially burdensome (Gardiner et al. 2020). Studies, including those by Morris et al. (2015) and Mulcahy Symmons et al. (2022), highlight the importance and challenges of caregiving, emphasizing the need for more support and resources.

Coordination, in the context of end-of-life care at home, involves synchronizing medical, emotional, and logistical support to provide comprehensive, patient-centered care. This includes clear communication between care teams, timely access to resources, and involvement of relevant professionals alongside family members to meet the patient’s needs and preferences. Liberati et al. (2016) examine how professional boundaries can hinder effective collaboration, thus increasing the need for coordination in complex care settings, while Buchan et al. (2019) discuss how the proliferation of roles in the National Health Service (NHS)has introduced more complexity, necessitating greater coordination to manage patient care effectively. While the need for coordination in healthcare may vary depending on system fragmentation, professional remits, or a transactional rather than relational care approach, this review highlights the intrinsic requirement for coordination in end-of-life care in all settings. This need exists to greater or lesser degrees, regardless of external factors. In many cases, family caregivers step into the role of coordinating care to ensure consistency and continuity, even when external agencies are involved. They often act as the central point of communication and logistical management, facilitating interactions between multiple care providers to support the patient’s comprehensive needs.

Rabow et al. (2004) present the paradox that families are expected to undertake complex physical caring tasks with little or no training and coordinate all aspects of care. More recent research has shown that coordination is a complex intervention and to be effective should involve all in the caring partnership and be resourced adequately (Davidson et al. 2015). The NHS describes the care coordinator position as helping to navigate health and social care systems, connecting people with the right teams, skilled in needs assessment and an “effective intervention” (Health Improvement Scotland 2019; NHS 2023). This emphasizes the necessity, significance and complexity of the role.

The experience of coordinating end-of-life care for a loved one can be an overwhelming and emotionally challenging task for family caregivers (Zhu et al. 2023). This review therefore aims to explore this critical element of home-based palliation.

## Aim

This review aimed to explore family caregiver experiences of coordinating end of life care in the home setting.

## Methods

### Search criteria

PICo (Population, phenomenon of Interest and Context) was utilized as a structured approach to framing the qualitative research question and to support the generation of keywords (Stern et al. 2014). The research question was

What are family caregivers’ (**P)** experiences of coordinating end of life care (**I**) in the home setting (**Co**)?

An initial search was conducted to establish whether this question had been addressed in previous systematic reviews and a crosscheck of the International Prospective Register of Systematic Review otherwise known as PROSPERO(National Institute for Health Research 2020) for currently unpublished reviews to prevent risk of duplication. A literature search was conducted using the “PICo” template to support the generation of relevant keywords (family caregiver experience, care coordination, end-of-life, palliative, home setting) and to provide structure for the search strategy and search terms: “Informal care* OR Family Care* OR Unpaid care* OR spous* OR relatives) AND Ti, ab (experience OR perspective*OR perception*OR ‘lived experience’ OR ‘personal experience’ AND Ti, ab (end of life* OR end of life care OR palliat* OR palliative care OR dying OR terminally ill) AND Ti, ab (coordinat*.” The key “PICo” themes were then used to identify appropriate synonyms, alternative spellings, and truncation. Five databases (CINAHL Plus, AMED, Medline, Cochrane Database of Systematic Reviews, and Joanna Briggs Institute for Systematic Reviews) were systematically searched from inception to 31 May 2023. Boolean operators were utilized to combine and refine the searches. The search details are available in Table 1.

**Table 1.** Search terms used for each database*: Keywords were* “*family caregiver,*” “*experience,*” “*end of life*” *and* “*coordination*” *and synonyms*

Database	Search terms and filters.	Hits
	Ti = title Ab = abstract Kw = keyword	
**CINAHL Plus**	Ti, ab (Informal care* OR Family Care* OR Unpaid care* OR spous* OR relatives) AND Ti, ab (experience OR perspective*OR perception*OR “lived experience” OR “personal experience” AND Ti, ab (end of life* OR end of life care OR palliat* OR palliative care OR dying OR terminally ill) AND Ti, ab (coordinat*) Filter: None	185
**Cochrane Database of Systematic Reviews**	Ti, ab, kw (Informal care* OR Family Care* OR Unpaid care* OR spous* OR relatives) AND Ti, ab, kw (experience OR perspective*OR perception*OR “lived experience” OR “personal experience” AND Ti, ab, kw (end of life* OR end of life care OR palliat* OR palliative care OR dying OR terminally ill) AND Ti, ab, kw (coordinat*) Filter: None	364
**AMED**	Ti, ab (Informal care* OR Family Care* OR Unpaid care* OR spous* OR relatives) AND Ti, ab (experience OR perspective*OR perception*OR “lived experience” OR “personal experience” AND Ti, ab (end of life* OR end of life care OR palliat* OR palliative care OR dying OR terminally ill) AND Ti, ab (coordinat*) Filter: None	137
**MEDLINE**	Ti, ab (Informal care* OR Family Care* OR Unpaid care* OR spous* OR relatives) AND Ti, ab (experience OR perspective*OR perception*OR “lived experience” OR “personal experience” AND Ti, ab (end of life* OR end of life care OR palliat* OR palliative care OR dying OR terminally ill) AND Ti, ab (coordinat*) Filter: None	212
**JBI – for systematic reviews**	Ti, ab (Informal care* OR Family Care* OR Unpaid care* OR spous* OR relatives) AND Ti, ab (experience OR perspective*OR perception*OR “lived experience” OR “personal experience” AND Ti, ab (end of life* OR end of life care OR palliat* OR palliative care OR dying OR terminally ill) AND Ti, ab (coordinat*) Filter: None	4

To increase the sensitivity of the search, additional manual search methods were employed, including search engine search via Google Scholar, and manual searches of reference lists from relevant articles (Boland et al. 2017).

The inclusion criteria sought studies that included adult carers or family caregivers over the age of 18 years caring for adult patients aged 18 years or over. Studies were included that explored the populations experience of coordinating care in the home setting. Study type included full text primary research available in English language, which included qualitative, mixed methods, case studies, and primary research in dissertations.

Studies were excluded that focused on child carers, child patients, or whether patients has an advanced terminal illness. Studies were also excluded that focused on the experience or perspectives other than those of family caregiver. Study context outside of the home setting as well as opinion pieces and published abstracts were also excluded.

The full inclusion and exclusion criteria are outlined in Table 2.

**Table 2.** Inclusion and exclusion criteria

	Inclusion criteria	Exclusion criteria
**Population**	Family caregivers (unpaid carers, informal carers) supporting people with a palliative diagnosis or at end of life Adult carers (age 18 years of over) or adult patients (age 18 years or over)	Formal care providers, child carers, child patients, unclear whether patients have advanced or terminal illness
**Interest**	Experiences of coordinating care	Perspectives other than those of family caregiver. Experience of other aspects of care. Burden of care.
**Context**	Home setting	Formal care settings; hospital, hospice, care homes
**Study design**	Primary research to include the following: mixed methods studies, qualitative studies, case studies, and primary research in dissertations. Available in English language, full text available	Opinion pieces, published abstracts

### Search outcomes and study selection

**Figure 1.**
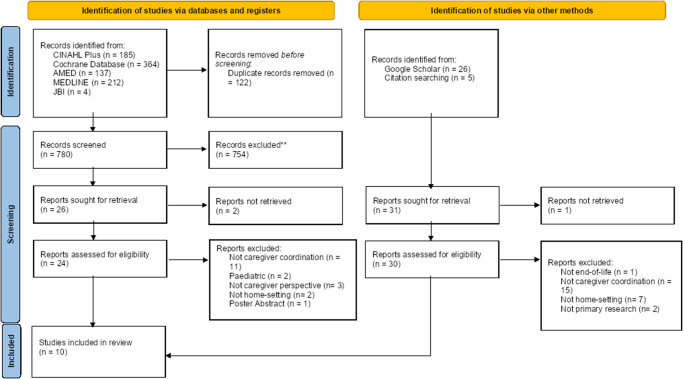
PRISMA flow diagram (adapted from Page et al., 2021). The PRISMA diagram below demonstrates the results of searches and screening.

A PRISMA flow diagram in Fig. 1 shows a summary of the search results. A total of 780 records were identified through the database searches, with a further 31 from Google Scholar and citation searches. Following initial screening a total of 26 full texts from databases and registers and 31 from other methods were retrieved to be assessed for eligibility. During full text screening, 47 papers were identified as not meeting the eligibility criteria and removed. A total of 10 studies were included in the review. A reference manager was used to assist in the initial screening process for organizing references and removing duplicates. In a 2-stage approach, titles and then abstracts were screened, to establish relevance. Those selected were then reviewed as full text in conjunction with the criteria for inclusion and exclusion. This selection process and the resulting articles for review was discussed and agreed with a second reviewer, to enhance reliability, minimize selection bias and add robustness to results (Butler et al. 2016). Any discrepancies were discussed with a third reviewer.

The potentially relevant studies were screened using the eligibility criteria, and then an iterative process ensued that involved revisiting the criteria and searching the content for “coordination.”

### Quality appraisal

The final selection for this review were all qualitative studies, therefore the focus for appraisal tool was to ensure robust qualitative approaches.

The JBI Critical Appraisal Checklist for Qualitative Research was used to evaluate the quality of each study chosen for the systematic review and findings discussed with another reviewer (Lockwood et al. 2024). All studies demonstrated good methodological quality.

### Data extraction

Data was collected in 2 steps, the first of which involved utilizing the JBI-QARI extraction tool (Aromataris and Munn 2020) to gather information from the chosen publications. The data were retrieved according to each study’s references, country of origin, methodology and data analysis, phenomena of interest, setting and participants, and author’s conclusions. See Table 3 for a description of the included studies.

**Table 3.** Extracted data of included studies

Study, year, country	Methodology, method and data analysis	Phenomenon of interest	Setting and participants (all had delivered care in home setting)	Author’s conclusions
Fenton et al. (**2023**): *United States of America – Massachusetts*	Qualitative – semi-structured interviews Codebook approach to thematic analysis Used NVivo11	To investigate cancer caregivers’ communication experiences and potential impact on patient and caregiver outcomes	Bereaved caregivers recruited through a cancer institute Relative had died within 2 years Purposive sampling *N* = 19, 14 female and 5 males, aged 40–82, mean age 64	Five categories were established to describe communication experience. Caregivers are responsible for complicated coordination of communications that facilitate goals of care. Challenges include avoidance by both patient and care teams in end-of-life planning. Coordination of discussions was emotionally draining, exacerbated by unclear communication from care professionals, including oncologists. If communication needs are met, caregivers can coordinate better care.
Hardy et al. (2014) – *England*	Qualitative – semi-structured interviews Thematic analysis using phenomenological techniques including template analysis. Heuristic method of interpretation	To explore how patients and spouse-carers manage their involvement with care professionals in the community setting.	Current caregivers and relative recruited through community nursing services. Purposive sampling *N* = 16, 8 pairs, all male–female, all aged 60–76 years	Authors presented 3 themes, illustrating spousal management. Spouse-carers are responsible for managing formalized care. They also pick up aspects of partner’s formers tasks e.g. finance management, domestic activity etc., which caused distress for patient. Strategies were developed – mapping out services and how to access them. Where strategies failed, they felt out of control. E.g. trying to obtain out of hours support over a weekend and struggling; the caregiver enlisted new strategy to aim for direct hospital admission if out of hours. Experiential learning, evolving strategies.
Linderholm and Friedrichsen (2010) – *Sweden*	Qualitative – interviews conducted with an interview guide Thematic analysis Heuristic method of interpretation	To explore how the informal carer of a dying relative, admitted to primary healthcare areas, experienced their caring role and support during the patient’s final illness and after their death	Four primary healthcare areas Bereaved caregivers recruited through district nursing Relative had died 3–12 months previously Purposive sampling *N* = 13, 8 females, 5 males, aged 38–78, mean age 58	Caregivers experienced their role as central, demanding great knowledge and requiring acknowledgment and reliable support. Important for caregivers to have opportunity to talk about their significant experiences. The overarching category was “a desire to be seen” with a number of subcategories that describe the coordinating experience.
Mohammed et al. (2018) – *Canada*	Qualitative – semi-structured interviews Thematic analysis Used NVivo 10 Grounded Theory – Corbin and Strauss methodology	To describe bereaved caregivers’ experiences of providing care at home for patients with advanced cancer, while interacting with home care services.	Bereaved caregivers recruited through a cancer center Relative had died 6 months–5 years previously Purposive sampling *N* = 61, 17 male, 44 females. Mean age 59.	Caregivers felt “thrust into this role” – a core category of taking charge – and were often uncomfortable. It was felt there was a lack of support, lack of information, and lack of training. Accessibility and consistency of staff were seen as valuable to coordinating care. Caregiver comments highlight inconsistency between the results of a “satisfaction survey” and lived experience.
Ojima Adejoh et al. (2021) – *Nigeria, Uganda and Zimbabwe*	Qualitative – conventional content secondary analysis of semi-structured interview transcripts Thematic analysis Used NVivo 12 Coding in alignment with the Consolidated Criteria for Reporting Qualitative Research (COREQ)	To understand the role, impact, and support of informal caregivers of patients with advanced cancer when interacting with palliative care services in Nigeria, Uganda, and Zimbabwe.	Current caregivers recruited through a parent study – participants from primary, secondary, and tertiary sites, reanalysis of data Purposive sampling *N* = 48, 24 male and 24 female aged 19–75 years, mean age 37	Informal caregivers coordinate matters of emotional health, physical health, and practical issues. They were balancing numerous priorities and identified the need for financial support and a need for key workers to provide continuity of care. This was true across all settings. The role of the caregiver as coordinator was central to the key themes.
Reeves et al. (2020) – *Switzerland*	Qualitative – semi-structured interviews Thematic analysis Used MAX QDA Corbin and Strauss methodology	To identify who plays a key role in coordination in palliative home care.	Current caregivers recruited through internet searches and personal referrals Purposive sampling *N* = 29 family caregiver (20 females, 9 males, mean age 59) *N* = 12 physicians *N* = 12 nurses	Core phenomenon found was the ambiguity of key coordinator role, and categories created looked at the causal conditions, context, consequences, and adopted strategies. Family caregivers were found to feel very responsible for the coordination of care, and vital to the processes. But perceptions differed e.g. HCP suggesting GP is the coordinator. Overarching was a lack of agreement as to who coordinated, but family caregiver experience was directly linked to this responsibility.
Salifu et al. (2021) – *Ghana*	Qualitative – semi-structured interviews Thematic analysis Social constructivist theory and interpretivism Used NVivo 12 Coding in alignment with COREQ	To explore palliative and end-of-life care experiences of family caregivers and patients living at home in a resource-poor context in Ghana.	Current caregivers recruited through an oncology department Purposive sampling *N* = 23 family caregivers *N* = 23 patients *N* = 12 healthcare professionals	Three main themes, with subthemes that overall establish the necessity for family caregivers to coordinate care. Resource-poor countries, with under-developed palliative care, have strong reliance on family caregiver, highlighting gap in care provision. While the answer isn’t a replica Westernized palliative model, better support for caregivers is required, such as telemedicine, and call for a compassionate community approach. Support networks for formal caregivers would be invaluable
Skorpen Tarbegr et al. (2019) – *Norway*	Qualitative – narrative interviews Inductive thematic analysis using Thompson’s framework	To explore how family caregivers experience involvement in palliative care	Bereaved caregivers recruited by oncology nurses in municipalities Relative had died 3–12 months previously Purposive sampling *N* = 11, 9 females, 2 males aged 31–80	Four interrelated themes derived from the narratives. Family caregivers experienced a lack of preparation and organized input from healthcare providers. Patients defined the need for information and healthcare providers colluded in this paternalistic approach, therefore coordinating the care was increasingly challenging. Patient-centered care can exclude the unmet needs of vital family caregivers and in doing so, undermine the coordination provision.
Totman et al. (2015) – *England*	Qualitative – semi-structured interviews Thematic analysis – interpretive phenomenological analysis Yalom’s model of existential conditions Used NVivo	To explore the emotional challenges faced by home caregivers, and their experiences of healthcare professionals, from the perspective of existential psychology.	Bereaved caregivers recruited through a London Hospice Relative had died approx. 3 months previously Purposive sampling *N* = 15, 11 females, 4 males, aged 27–64	Fifteen themes that were categorized into Yalom’s framework of 4 existential conditions. Found that relatives took on high levels of responsibility. Perceived failures in care coordination were identified and highlights the risks of family having to advocate. Responsibility and coordinating care were uniquely intense. And driven by the need to “get it right.” Carers questioned whether they were doing things right or doing enough, feeling anxious and isolated. Feeling unsupported can intensify the burden of responsibility and isolation.
Vermorgen et al. (2021) – *Belgium*	Qualitative – semi-structured interviews Interpretative phenomenological analysis Used NVivo 11	To investigate how family carers of people who live at home with a life-limiting chronic illness experience and perceive collaboration with different healthcare professionals in the last phase of life	Current caregivers recruited through treating physicians in different disease specialities Purposive sampling *N* = 30 4 male, 26 females. aged,45–75	Five major themes, overall identifying caregiver as key coordinator. Informal caregivers felt disregarded as experts by HCPs, not treated as members of the team. Being able to contact services once *invited* was an important aspect of coordinating the care, alongside consistency of HCP. Jargon also hindered true care partnership. “in the dark” about social care. Perceived missed opportunities for HCPs to collaborate effectively.

### Synthesis

Data was thematically extracted, using manual open coding. A meta-aggregative approach was used for data synthesis by pooling the findings together and then grouping these into categories according to their meaning. Categories were then further paired into synthesized themes. As the studies selected were comparable and contain some defined themes, deductive integrative synthesis was considered the most appropriate and adopted for the review (Boland et al. 2017). Any discrepancies in the synthesis process were discussed with other members of the team. Manual coding enabled a reflective approach to the data, and reflexivity was maintained through critical reflection on assumptions and biases that may have been introduced.

## Results

### Characteristics of the studies

A total of 10 studies met the inclusion criteria, all were published between 2010 and 2023. The search resulted in studies from Africa, USA, Canada, England, Belgium, Switzerland, Sweden, and Norway. Most of these studies (*n* = 8) deployed a semi-structured interview approach to obtaining the data, also secondary analysis of semi-structured interviews (*n* = 1) and narrative interview (*n* = 1). Qualitative methodological approaches used included grounded theory, a phenomenological approach, a heuristic method, and social constructivist theory. Studies had between 11 and 61 caregiver participants, a number congruent with the methodology of the studies (Creswell and Creswell 2018). A total of 245 adult family caregivers reported experiences of coordinating end-of-life care, ages ranging from 19 to 82 years of age. Analyses of the data collected included inductive thematic analysis, interpretative phenomenological analysis, Heuristic interpretation, and Corbin and Strauss methodology.

## Review findings

A total of 88 findings were located. These were further aggregated into 12 categories and then synthesized into 3 overall themes: (1) family caregiver identity (2) strategies for care, and (3) practicalities of care, as presented in Table 4.

**Table 4.** Synthesized findings

Synthesized finding	Categories	Findings
***Family caregiver identity***	Terminology of role	The family caregiver self-identifies as the “coordinator” (Reeves et al. 2020) The caregiver is “part of the team” (Vermorgen et al. 2021) Becoming a “take charge person” (Mohammed et al. 2018), being “main” or “only” carer (Hardy et al. 2014; Ojima Adejoh et al. 2021) or playing “middleman” (Fenton et al. 2023). Totman et al. (2015) described the family caregiver as “being the linchpin,” while a relative described his wife as “my doctor at home” (Salifu et al. 2021). (8 findings)
	Relationship with their relative and relative’s former role	Caregivers take on the relative’s former tasks, such as finance and domestic management (Hardy et al. 2014; Totman et al. 2015). They are also supporting the relative’s emotional needs and feelings including loss of independence (Fenton et al. 2023; Hardy et al. 2014; Ojima Adejoh et al. 2021; Reeves, Liebig and Schweighoffer; Salifu et al. 2021; Skorpen Tarbegr et al. 2019; Totman et al. 2015) (9 findings)
	End-of-life care coordination is not optional, it is an extension of the relationship	Caregivers described the moral imperative and social obligation to undertake the coordinating role for their relative (Hardy et al. 2014; Linderholm and Friedrichsen 2010; Mohammed et al. 2018; Reeves et al. 2020; Salifu et al. 2021) And they were not part of the decision (Skorpen Tarbegr et al. 2019) Some felt this was a reciprocity, their turn to care for their loved one (Ojima Adejoh et al. 2021; Totman et al. 2015) (8 findings)
	Caregiver’s feelings of anxiety, isolation and loneliness when coordinating end-of-life care	Caregivers described the feeling of responsibility as frightening and induced anxiety (Salifu et al. 2021; Skorpen Tarbegr et al. 2019) They also discussed feeling isolated, lonely, or alone in their burden (Linderholm and Friedrichsen 2010; Totman et al. 2015), going so far as using search engines to find out what to do (Fenton et al. 2023) (5 findings)
	Rewarding experience	Successful care coordination gave caregivers a sense of control and safety (Hardy et al. 2014) and a sense of usefulness (Reeves et al. 2020). Having the opportunity to provide this care was a blessing in disguise (Ojima Adejoh et al. 2021), or a privilege (Totman et al. 2015) (4 findings)
Synthesized finding	Categories	Findings
***Strategy for care***	Approach and planning	Family caregivers developed strategies for coordinating care, such as mapping out the community services (Hardy et al. 2014) and developing new strategies when something failed (Hardy et al. 2014). They independently acquired knowledge (Fenton et al. 2023; Reeves et al. 2020), used a notebook system (Linderholm and Friedrichsen 2010; Vermorgen et al. 2021), assertively accessed senior people on the phone (Mohammed et al. 2018) Caregivers used hospital admission as a strategy to manage complex situation and were unable to access support (Hardy et al. 2014; Salifu et al. 2021) (9 findings)
	Decision-making	Decision-making responsibilities were central to the coordination of care and identified by many caregivers as a key duty (Fenton et al. 2023; Hardy et al. 2014; Mohammed et al. 2018; Reeves et al. 2020; Salifu et al. 2021; Totman et al. 2015) (6 findings)
	Continuity	Family caregivers expressed that they were providing continuity as an element of coordinating the care (Mohammed et al. 2018; Reeves et al. 2020) and that continuity from the healthcare professionals enabled the family caregiver to better coordinate the care (Ojima Adejoh et al. 2021; Vermorgen et al. 2021) (4 findings)
	Communication and liaison	Another key aspect described by caregivers was communication and liaison between themselves, the patient and healthcare service (Fenton et al. 2023; Ojima Adejoh et al. 2021; Reeves et al. 2020; Vermorgen et al. 2021) and this could be very time-consuming for them, especially on the phone (Mohammed et al. 2018; Totman et al. 2015). It could also involve interpreting and translating, either language or jargon or implicit meanings (Fenton et al. 2023; Ojima Adejoh et al. 2021) (8 findings)
Synthesized finding	Categories	Findings
***Practicalities of care***	Symptom and medicines management	Caregivers are required to assess the significance of symptoms and manage medications (Hardy et al. 2014; Reeves et al. 2020). Sometimes this is an unspoken expectation from the relative (Linderholm and Friedrichsen 2010) and feeds back into the role of decision-maker (Mohammed et al. 2018), sometimes it is a joint process between the caregiver and the patient (Salifu et al. 2021), and on occasion it is a source of distress for the patient if it is something personal, such as catheter management (Hardy et al. 2014). The caregiver will ask the relative about their pain and encourage them to take medication if they have access to pain relief (Ojima Adejoh et al. 2021), they will also judge the symptom burden in relation to accessing health services (Ojima Adejoh et al. 2021). In a resource-poor setting, access to pain medication is limited and results in caregivers trying to coordinate symptom control and turning to alternative options (Salifu et al. 2021). (9 findings)
	Equipment management	Alongside symptom management, caregivers coordinate the need for and access to equipment for the home and relative (Fenton et al. 2023). In a resource-poor setting, this may include raising funds and managing without needed items, such as stoma bags (Ojima Adejoh et al. 2021). It was necessary to arrange equipment but also send back when inappropriate (Mohammed et al. 2018). Being unable to access the necessary equipment can cause distress (Totman et al. 2015) (4 findings)
	Care appointments, services management and advocacy	In coordinating care, caregivers organize and manage appointments (Hardy et al. 2014). They know about their relative’s treatments and attend appointments (Reeves et al. 2020). Appointments may require the caregiver to advocate for their relative to maintain well-being (Fenton et al. 2023). Caregivers negotiate which healthcare professionals are accepted or refused in the home setting (Mohammed et al. 2018). Sometimes caregivers attend appointments on behalf of their relative (Ojima Adejoh et al. 2021). Some experienced a sense of embattlement when dealing with other services on behalf of their relative (Totman et al. 2015). (14 findings)

### Family caregiver identity

Five categories were included in the first synthesized theme. “Terminology of role” demonstrated that caregivers self-identified as the coordinator (Hardy et al. 2014; Mohammed et al. 2018; Reeves et al. 2020; Vermorgen et al. 2021). Some caregivers felt they played an intermediary role in coordinating care (Fenton et al. 2023).

“Relationship with their relative and relative’s former role” incorporated supporting the relative’s emotional needs (Fenton et al. 2023; Totman et al. 2015). These findings included loss of the relative’s role and independence (Ojima Adejoh et al. 2021). Caregivers also addressed the change in their relationship (Skorpen Tarbegr et al. 2019).

“End-of-life care coordination is not optional; it is an extension of the relationship” included not being part of the decision (Linderholm and Friedrichsen 2010; Skorpen Tarbegr et al. 2019; Reeves et al. 2020; Ojima Adejoh et al. 2021). Also addressed is the moral imperative, articulated as feeling obligated or having spiritual repercussions if family did not provide care (Mohammed et al. 2020; Salifu et al. 2021). Some caregivers felt this was a reciprocity for care they had themselves been given (Salifu et al. 2021; Totman et al. 2015).

“Caregiver’s feelings of anxiety, isolation and loneliness when coordinating end-of-life care” related to the feeling of responsibility causing anxiety (Linderholm and Friedrichsen 2010; Salifu et al. 2021; Skorpen Tarbegr et al. 2019; Totman et al. 2015).

“Rewarding experience” highlighted that while many of the categories showed responsibility and burden for family caregivers, they also reported the positive aspects to this role or an opportunity to demonstrate better care than they themselves had previously received (Totman et al. 2015; Ojima Adejoh et al. 2021; Salifu et al. 2021).

### Strategy for care

Four categories were identified in the second synthesized theme. “Approach and planning” identified that family caregivers developed their own strategies for coordinating the care for their relative. This included mapping out the services available to them and developing something new when previous strategies failed (Hardy et al. 2014). Obtaining information independently was another approach (Fenton et al. 2023; Reeves et al. 2020). Some caregivers described using a written system to strategize (Linderholm and Friedrichsen 2010; Vermorgen et al. 2021). Caregivers also strategized hospital admission to manage complex situations (Hardy et al. 2014; Salifu et al. 2021).

Responsibility for, and effectiveness in, “decision-making” was central to the coordination role for caregivers. This could include choices around the treatment (Fenton et al. 2023). Caregivers described the responsibility that came with this aspect of the role (Mohammed et al. 2018; Reeves et al. 2020).

Caregivers expressed their provision of “continuity” as part of the coordinating role (Mohammed et al. 2018; Reeves et al. 2020). There was recognition from the caregivers that continuity from the healthcare professionals would have enabled them to coordinate care more easily (Ojima Adejoh et al. 2021; Vermorgen et al. 2021).

“Communication and liaison” related to the caregiver’s responses around communicating with others involved in the care of their relative, and the relative themselves, plus other family. Caregivers discussed managing difficult communication with the healthcare teams, family or with their relative about dying and death (Fenton et al. 2023; Mohammed et al. 2018). When discussion about dying did happen, it was profoundly helpful for the caregiver (Mohammed et al. 2018). Communication and liaison were a heavy workload for caregivers (Hardy et al. 2014; Totman et al. 2015). This aspect of the role could also involve translation, of language or jargon, to support the coordination of care (Ojima Adejoh et al. 2021; Fenton et al. 2023).

### Practicalities of care

Three categories were identified in the third synthesized theme. “Symptom and medicines management” indicated that caregivers found themselves being responsible for assessing symptoms and managing medications (Hardy et al. 2014; Reeves et al. 2020). This element of coordination could feed back into decision-making (Mohammed et al. 2018) or be a joint process between the patient and the caregiver (Ojima Adejoh et al. 2021). In some cases, caregivers reported the need to judge severity in relation to accessing further help (Ojima Adejoh et al. 2021). In resource-poor settings, access to pain medication could be limited, resulting in caregivers trying to manage poor symptom control and turning to alternative options (Salifu et al. 2021).

“Equipment management” was another area that caregivers found themselves coordinating (Fenton et al. 2023). In a resource-poor setting, the added complexity could include raising funds and managing without necessary items (Ojima Adejoh et al. 2021) and being unable to access necessary equipment was a cause of distress (Totman et al. 2015).

“Care appointments, services management and advocacy” was exhibited by, for instance, the caregivers attending the appointments with the relative (Reeves et al. 2020), or even on behalf of them (Ojima Adejoh et al. 2021). The caregiver may advocate for their relative in these situations (Fenton et al. 2023). Caregivers reported experiences of negotiating which healthcare professionals would be involved (Hardy et al. 2014), and there were repeated reports of general management of services (Mohammed et al. 2018; Salifu et al. 2021; Skorpen Tarbegr et al. 2019). Some of these interactions were difficult and gave a sense of embattlement to coordinating (Totman et al. 2015). One caregiver identified an action that could be taken to support them (Ojima Adejoh et al. 2021).

## Discussion

The themes of this narrative review demonstrate the many facets of coordinating end-of-life care for family caregivers. Interestingly, none of the studies focused purely on this aspect of care provision. It therefore highlights that while the subject of coordinating care, especially in the end-of-life context, is a relevant and documented topic, there has not been a defined focus on the family caregiver’s responsibilities in this. The findings draw attention to the role of family caregiver as contextual and subjective to the person(s). This potential subjective response is reflected in other studies that explore family caregivers’ experiences, albeit not identifying coordination as a role. Carlander et al. (2011)illustrated the sense of main responsibility that this cohort feels for their relative, while Woodman et al. (2016) describe the feelings of obligation family caregivers to enable care in the home setting. The choice for place of care and death, while patient-centered, should involve the family caregiver. This choice should be open to reevaluation if the situation changes, due to the risks of placing such remit on that 1 caregiver (Fereidouni et al. 2021; Munck et al. 2008).

In the same connotation as people not identifying themselves as a carer at all (The National Institute for Health and Care Excellence 2020), the relationship with the relative was a “mixed blessing.” The sense of obligation and lack of choice was made clear in 8 out of the 10 studies, but alongside this the expression of close bond with the relative. It is unsurprising that healthcare professionals make assumptions of family willingness to provide home-based care when patients are non-curative and have limited treatments (Linderholm and Friedrichsen 2010; Mohammed et al. 2018).

Findings also suggest that inadequate preparation for signs and symptoms that may appear near death, caused distress impacting facilitating the coordination of care. Communication about the dying process helps the family caregivers be prepared for what is happening and what is to come (Robertson et al. 2022). Caregivers felt that healthcare professionals did not provide them with the information they required, and evidence supports the view that family caregivers’ informational needs are not being met (Woodman et al. 2016; Zhu et al. 2023).

Family caregivers adapt their strategies with a trial-and-error process to cultivate their own remedies and use experiential learning, especially when navigating services, a tactic corroborated in other studies (Michaels et al. 2021). Hospitalization is used as a further strategy when services are not felt to be meeting the relative’s needs in the home setting. This suggests that despite developed policy and palliative care provision, there remains a gap in the ideal delivery of support.

Communication was a key coordination activity and a challenge identified by caregivers. The impact of being compelled to repeatedly seek clarity from healthcare professionals and at times being excluded from discussions when it was felt that they were key to understanding their relative and circumstances (Martín et al. 2016). Exclusion bled into frustration and feeling discounted or even ignored during healthcare interactions with the relative (Linderholm and Friedrichsen 2010; Skorpen Tarbegr et al. 2019). Family caregivers having successful communication is essential to coordinating care and where this can be supported or improved demonstrates a more fulfilled role.

The findings highlighted how family care givers were responsible for providing practical care, including assessing and managing symptoms (Morris et al. 2015), which in the absence of professional help may result in emergency hospital admission. Morris et al. (2015) concluded correspondingly, discussing the multifaceted interchange between resources and family caregivers that impacts the perception of their experience.

It is further complicated in resource-poor settings where medication and equipment are not as readily prescribed and the weight of responsibility falling on them alone. The international implications of this narrative review highlight the universal challenges and variations in family caregiving for end-of-life care at home. Cultural, economic, and healthcare system differences across countries shape the resources and support available to caregivers. In low- and middle-income countries, caregivers often face limited access to professional healthcare services and palliative care, intensifying their burden. In contrast, high-income nations may offer more comprehensive healthcare infrastructure, yet disparities in caregiver support persist. Understanding these international contexts can inform global health policy, promoting equitable access to resources, training, and support for family caregivers worldwide, regardless of geographic or socioeconomic boundaries.

## Limitations

To the best of the author’s knowledge, this is the first narrative review focusing exclusively on the family caregiver experience of the coordination role when a relative is receiving end-of-life care. The search strategy was limited to 5 databases, alongside reference searches and Google Scholar, which could have limited the number of studies included. Unintentional study exclusion could also have been exacerbated by the chosen search terms and Boolean operators, particularly because some of the concepts were nonspecific in terminology. For instance, “end-of-life” and synonyms – other terms could have been used by study authors, such as “Hospice.” Similarly, searching the term “coordination” within records could potentially have excluded papers that defined this activity in another way. It is also worth noting that using 3 elements, instead of 4 in the search strategy may have given a higher yield. Furthermore, the author is a nurse practitioner working within the area of palliative care and therefore well-placed to understand the concepts but at risk of introducing bias and preconceived beliefs and conclusions. Lastly, several of the studies used participants who were bereaved caregivers, and this can introduce recall bias in their reports.

However, the review methodology was systematic and comprehensive, with a meta-aggregative approach to synthesizing the findings. The reliability of the review is improved by this iterative process of identification and could be considered transferable.

## Implications for research and practice

In future research, several areas warrant further exploration to enhance understanding of the challenges family caregivers face. For instance, the loss of relationship and independence, as highlighted in the theme family caregiver identity, should be examined more deeply to determine its emotional and psychological impact on caregivers. Furthermore, from the same theme, there is potential to explore how anxiety, loneliness, and isolation among caregivers can be mitigated through access to personal support services.

The concept of caregiver decision-making and empowerment should be explored further, particularly in relation to how education and training programs might provide support. Future research should also examine how these initiatives can be implemented and adapted on a global scale to address diverse caregiving needs across different cultures and healthcare systems.

Additionally, an investigation of cultural, religious, and spiritual obligations, which vary across countries, may help healthcare providers to understand how they shape caregiving experiences internationally.

Recommendations for policy makers are 2-fold: 1 is the need to address the obligation of family care provision. This can be achieved by subscribing to an enhanced communication agreement through a central care provider, such as a General Practitioner (GP)or specialist nurse, that safeguards the choices and discussions, so they are inclusive of the family caregiver in addition to the patient. The other component is reviewing the current status of family care provision, with aim to make it less burdensome and more rewarding. This should include ensuring there is a contingency plan for patients that wish to be cared for at home. The plan needs to include availability of social care domiciliary support that enables home-based care without a family caregiver present or, arguably more sustainable and satisfactory, enabling increased support for the family caregiver.

## Conclusion

Coordinating end-of-life care involves numerous challenges for family caregivers, including feelings of duty and isolation, and communication issues. These difficulties are inherent in the role of being a primary caregiver. Care coordination becomes inevitable as caregivers are involved in tasks such as symptom assessment, liaising with healthcare professionals, and providing holistic support.

Evidence suggests that having a consistent professional contact, such as a palliative care facilitator, can help to distribute responsibilities and improve communication. This support would reduce caregivers’ anxiety, alleviate feelings of isolation, and potentially prevent unnecessary hospital admissions. Simplifying care through a key professional role or service should enhance the patient experience and improve outcomes for both caregivers and the healthcare system.

It should be stated that there is a need for more research on the unique challenges faced by family caregivers in coordinating end-of-life care for patients with life-limiting conditions, especially nonmalignant disease. However, it is unclear what this would add at this stage – the concerns and challenges faced by home-based palliative care are becoming apparent and this review of coordination experience adds to that body of evidence. Future studies should focus on the impact of facilitator roles and effective communication strategies, with potential international applicability.

Whilst some countries do not have the same degree of policy development, and certainly limited by reduced access to resources, they could also learn from and improve upon the attempts made by countries that have included palliative care on their health policy agenda for several years.
